# Transactive response DNA-binding protein-43 proteinopathy in oligodendrocytes revealed using an induced pluripotent stem cell model

**DOI:** 10.1093/braincomms/fcab255

**Published:** 2021-10-26

**Authors:** Samantha K Barton, Dario Magnani, Owen G James, Matthew R Livesey, Bhuvaneish T Selvaraj, Owain T James, Emma M Perkins, Jenna M Gregory, Elaine Cleary, C Rosanne M Ausems, Roderick N Carter Carter, Navneet A Vasistha, Chen Zhao, Karen Burr, David Story, Alessandra Cardinali, Nicholas M Morton, Giles E Hardingham, David J A Wyllie, Siddharthan Chandran

**Affiliations:** 1 Euan MacDonald Centre for MND, University of Edinburgh, Edinburgh EH16 4SB, UK; 2 Centre for Clinical Brain Sciences, University of Edinburgh, Edinburgh EH16 4SB, UK; 3 UK Dementia Research Institute at University of Edinburgh, University of Edinburgh, Edinburgh, EH16 4SB, UK; 4 Florey Institute of Neuroscience and Mental Health, Melbourne 3052, Australia; 5 Centre for Discovery Brain Sciences, University of Edinburgh, Edinburgh EH8 9XD, UK; 6 Department of Neuroscience, SITraN, University of Sheffield, Sheffield S10 2HQ, UK; 7 Centre for Cardiovascular Science, University of Edinburgh, Edinburgh EH16 4SB, UK; 8 Centre for Brain Development and Repair, Institute for Stem Cell Biology and Regenerative Medicine, Bangalore 560065, India

**Keywords:** amyotrophic lateral sclerosis, oligodendrocytes, TDP-43, induced pluripotent stem cell

## Abstract

Oligodendrocytes are implicated in amyotrophic lateral sclerosis pathogenesis and display transactive response DNA-binding protein-43 (TDP-43) pathological inclusions. To investigate the cell autonomous consequences of TDP-43 mutations on human oligodendrocytes, we generated oligodendrocytes from patient-derived induced pluripotent stem cell lines harbouring mutations in the *TARDBP* gene, namely G298S and M337V. Through a combination of immunocytochemistry, electrophysiological assessment via whole-cell patch clamping, and three-dimensional cultures, no differences in oligodendrocyte differentiation, maturation or myelination were identified. Furthermore, expression analysis for monocarboxylate transporter 1 (a lactate transporter) coupled with a glycolytic stress test showed no deficit in lactate export. However, using confocal microscopy, we report TDP-43 mutation-dependent pathological mis-accumulation of TDP-43. Furthermore, using *in vitro* patch-clamp recordings, we identified functional Ca^2+^-permeable α-amino-3-hydroxy-5-methyl-4-isoxazolepropionic acid receptor dysregulation in oligodendrocytes. Together, these findings establish a platform for further interrogation of the role of oligodendrocytes and cellular autonomy in TDP-43 proteinopathy.

## Teaser

Barton et al. report that oligodendrocytes derived from patient-induced pluripotent stem cells that harbour mutations in the *TARDBP* gene (these mutations are causative of amyotrophic lateral sclerosis) exhibit intrinsic dysfunction. They harbour pathogenic protein accumulation as well as have altered α-amino-3-hydroxy-5-methyl-4-isoxazolepropionic acid receptor expression, compared to control oligodendrocytes.

## Graphical Abstract

**Graphical Abstract:**
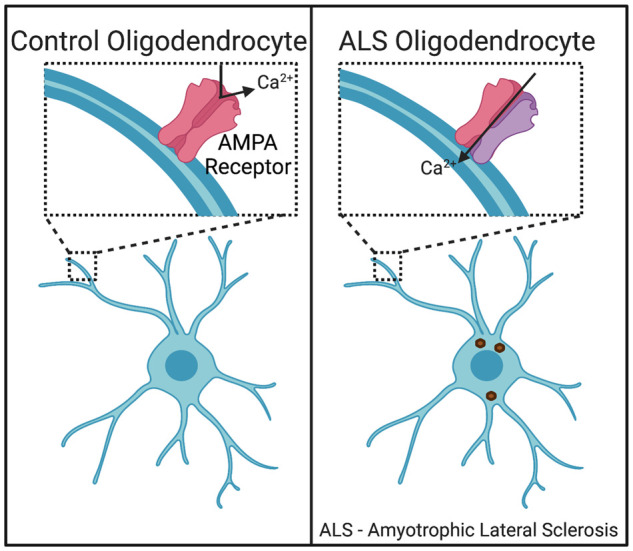

## Introduction

Neuronal and glial transactive response DNA binding protein-43 (TDP-43) inclusions are the pathological signature common to ∼95% of ALS and ∼50% of frontotemporal lobar degeneration cases. This includes disease due to a wide range of genetic mutations [*TARDBP* (encodes TDP-43 protein)*, PGRN, UBQLN2, SQSTM1, PFN1, ANG, VCP, MATR3, TUB4A and C9ORF72*], which implicates shared disease mechanisms and a causative role for TDP-43 proteinopathy.^1^ A recent study using a transgenic TDP-43 mouse model demonstrated that whilst mutant TDP-43 in neurons was necessary for symptom onset, disease progression continued even after neuronal TDP-43 expression was reduced suggesting a strong pathogenic role for the surrounding glia.^2^ Until recently, the focus of non-cell autonomous mechanisms in ALS has been on the role of astrocytes^3^^,^^4^ and microglia.^5^^,^^6^

Studies in post-mortem samples have demonstrated TDP-43 positive inclusions in oligodendrocytes, and a range of experimental and pathological studies have implicated oligodendrocyte lineage cells in the pathogenesis of ALS.^7–11^ These studies highlight both cell and non-cell autonomous processes.^12–15^ Furthermore, we, and others, have reported a subset of ALS patients who display exclusively oligodendroglial TDP-43 inclusions with no detectable neuronal TDP-43 proteinopathy.^12^^,^^16^ Animal studies have shown the critical importance of normal TDP-43 function in oligodendrocytes whereby selective deletion of *TDP-43* from oligodendrocytes in mice causes motor impairments and changes to myelination.^17^ Along with the finding in a TDP-43^Q331K^ ALS mouse model of upregulation in expression of myelination genes correlating with altered behaviour,^18^ these studies collectively implicate an important role for oligodendrocytes in ALS. Further, in demyelinating diseases, a key pathway driving oligodendrocyte injury is excitotoxicity.^19^ Excitotoxicity is a well-established pathway in ALS with an association between TDP-43 pathology and α-amino-3-hydroxy-5-methyl-4-isoxazolepropionic acid receptor (AMPAR) dysregulation.^20^ Our previous work has shown iPSC-derived motor neurons harbouring an expansion repeat mutation in the *C9orf72* gene have a dysregulated AMPAR phenotype in favour of Ca^2+^-permeable AMPARs leading to an increase in vulnerability to excitotoxicity. Further, there is regional and cellular heterogeneity of AMPAR dysregulation in ALS, with these data spanning iPSC models as well as human post-mortem tissue samples.^21–23^ Whether AMPAR dysregulation is present within oligodendrocytes exhibiting TDP-43 pathology remains to be determined. Any disruption to oligodendrocyte homeostasis could have detrimental effects on key functions such as myelin maintenance and metabolic capacity, both of which are critical for axonal health and function.

Here, we report the establishment and characterization of a human model to examine the cell autonomous consequences of *TARDBP* mutations in oligodendrocytes using two patient-derived iPSC lines each harbouring single point mutations in the *TARDBP* gene (G298S and M337V). Herein, we present a comprehensive assessment of development, morphology and function of oligodendrocytes harbouring mutations in the *TARDBP* gene, compared to appropriate controls. We show TDP-43 pathology in iPSC-derived oligodendrocytes, which is reversed in a CRISPR-Cas9 gene-edited control. Further, we identify a dysregulation in AMPARs in TDP-43^G298S^ oligodendrocytes. Our data support a genotype-specific cell autonomous impairment in oligodendrocytes and establish a platform for further mechanistic studies.

## Materials and methods

### Induced pluripotent stem cells generation, maintenance and gene editing

Three patient-derived iPSC lines were used; six lines in total: one control line (Control), two clones from one *TARDBP*^G298S^ patient line (ALS1^G298S^ and ALS2^G298S^; male, 64 years old), two gene corrected lines from ALS2^G298S^ (ALS2^G298S^-corr1 and ALS2^G298S^-corr2) and one clone from one *TARDBP*^M337V^ patient line (ALS^M337V^; male, 59 years old). The derivation, characterization and validation of iPSC lines were performed as described.^24^

### CRISPR/Cas9 gene editing in induced pluripotent stem cells

CRISPR/Cas9 mediated gene editing was performed on ALS2^G298S^ iPSC clonal line. U6 promoter followed by gRNA (5′-GCTAGTTTGGGAAACAATCA-3′) and gRNA scaffold was cloned into a pMTL23 vector through *HindIII* and *Xho1* restriction site cloning. ALS2 iPSC line was dissociated using 1× Accutase (Sigma); 8 × 10^5^ cells were nucleofected with 0.5 µg of pMTL23-gRNA, 1.5 µg of pSpCas9-2A-GFP (px458) plasmid and 100 pmol ssODN (180 bp repair template) using the Amaxa 4D nucleofector system (program CA137) following the manufacturer’s instructions. Following transfection, cells were plated on Matrigel (BD)-coated dishes in E8 medium supplemented with ROCK inhibitor (10 µM). Once cells reached confluency, they were again dissociated into a single cell suspension and plated at low density for clonal analysis. Each colony was screened for the accurate homologous recombination resulting in the replacement of the guanine at ORF position 892 with an adenine resulting in a gene correction from serine to glycine. The success rate of the CRISPR-Cas9 process was 0.7% and two colonies were expanded and maintained for use in experiments; one (ALS2^G298S^-corr1) had biallelic correction and the other (ALS2^G298S^-corr2) had monoallelic correction. By utilizing the two clones, we could guarantee that results were not due to CRISPR intervention and were a real result of the *TARDBP*^G298S^ gene correction.

### Oligodendrocyte generation from induced pluripotent stem cells

The patterning and derivation of oligodendrocytes from iPSC has been extensively described previously.^22^^,^^25^,^27^ Briefly, iPSC were lifted into suspension and underwent neuralization and caudalization. Neurospheres containing OLIG2+ cells were treated with FGF2 (10 ng/mL), PDGF-AA (platelet-derived growth factor AA, 20 ng/mL, PeproTech), purmorphamine (1 μM, Sigma), SAG (1 μM, Calbiochem), IGF-1 (insulin-like growth factor-1, 10 ng/mL, PeproTech), heparin (5 µg/ml), T3 (triiodothyronine, 45 ng/mL, Sigma) to selectively pattern for oligodendrocytes. Terminal differentiation of oligodendrocytes was achieved by dissociation using the Papain Dissociation System (Worthington Biochemical) and plated on coated plates [Matrigel (BD Biosciences), Laminin (Sigma) and Fibronectin (Sigma)]; at this stage, 1 x ITS (Insulin-Transferrin-Selenium, Gibco) was added and all mitogens were removed except for T3 and IGF-1. A minimum of three independent differentiations were conducted per line for analyses.

### Electrophysiology

To identify and visualize OPCs and oligodendrocytes within the cultures, OPCs and oligodendrocytes were stained live using PDGFRα or O4 antibodies and subsequently labelled with secondary Alexa 488 or Alexa 555 antibodies, respectively, prior to recording. The whole-cell patch configuration was used to record macroscopic currents as previously described.^26^ Current and voltage measurements were typically low-pass filtered online at 2 kHz, digitized at 10 kHz via a BNC-2090A (National Instruments) interface, and recorded to computer using the WinEDR V2 7.6 (J. Dempster, Department of Physiology and Pharmacology, University of Strathclyde, UK). Assessment of currents evoked by AMPAR were measured as detailed previously.^23^

### Quantitative RT-PCR

RNA was isolated from MACS-sorted O4-positive oligodendrocytes by using the RNeasy kit (Qiagen), following the manufacturer’s instructions. DNA contaminants were eliminated by using the Turbo DNA-Free kit (Invitrogen). cDNA was synthesized from 0.5 μg of total RNA with the DyNAmo cDNA Synthesis Kit (Thermo Scientific) in a 20 μL volume. Real-time quantitative polymerase chain reactions were performed in triplicate by using the DyNAmo Flash SYBR Green quantitative polymerase chain reactions kit (Thermo Scientific) and examined on a CFX96 System (Bio-Rad). Relative expression levels were calculated by the ΔΔCt method with GAPDH and 18S as the reference genes. Primer sequences used were:

**Table:** 

**GAPDH_F**	5′-GAG TCC ACT GGC GTC TTC AC
**GAPDH_R**	5′-ATG ACG AAC ATG GGG GCA T
**18S_F**	5′-GTA ACC CGT TGA ACC CCA TT
**18S_R**	5′-CCA TCC AAT CGG TAG TAG CG
**TDP-43_F**	5′-CGG CCT AGC GGG AAA AGT AAA AGA
**TDP-43_R**	5′-AGC ACC GTC CCA TCG TCT T
**MCT1_F**	5′-GAC CTT GTT GGA CCC CAG AG
**MCT1_R**	5′-AGC CGA CCT AAA AGT GGT GG

### Immunocytochemistry

All steps were performed at room temperature. Cells were fixed with 4% paraformaldehyde in PBS for 15 min, permeabilized with 0.3% Triton X-100 containing PBS and then blocked in 5% goat serum. This was followed by incubation with appropriate primary (TDP-43, Abnova #H00023435-M01, 1:200; Olig2, Millipore #AB9610, 1:200; PDGFRα, Cell Signalling #5241S, 1:200; O4, R&D Systems #MAB1326, 1:500; MBP, Millipore #AB7349, 1:50) and secondary (Alexa Fluor dyes; Invitrogen, 1:1000) antibodies. The nuclei were counterstained with DAPI (Sigma) and coverslips were mounted on slides with FluorSave (Merck).

### Semi-quantitative densitometry analysis and Sholl analysis

Cells were imaged with an Axioscope (Zeiss) microscope or LSMZ10 Confocal microscope and processed with Axiovision v 4.8.1 (Zeiss). Semiquantitative immunofluorescence analysis was performed using ImageJ64 (v1.45, NIH). Fields were selected based on uniform DAPI staining and imaged in three channels. For the analysis, images were first converted to grayscale and the DAPI and MBP channels were used to create regions of interest for quantification of cells harbouring granular TDP-43 cytoplasmic staining. Cell counts (Olig2, and PDGFa and MBP as a proportion of O4) were done manually using ImageJ64 (v1.45, NIH). Sholl analysis was performed on MBP^+^ cells using ImageJ with a starting radius 10 μm, step size 2 μm and sampled five times per radius.

### Flow cytometry

Flow cytometry of oligodendrocytes to ascertain the population of O4-positive cells was performed on a FACSCalibur (Becton Dickinson, San Jose, CA). Cells were lifted with Accutase (Sigma) and stained with primary antibody O4 1:500 (R&D Systems; #MAB1326) and then secondary IgM (see above) 1:3000. Secondary and unstained controls were also performed. The cells were analysed by forward and side scatter for Alexa Fluor 488 fluorescence through a 530 ± 30 nm band-pass. Unstained cells were used to set the background fluorescence; a false positive rate of 0.5% was accepted. FACS data were collected and analysed using Cellquest and Flowjo software.

### Glycolytic stress test

Following papain dissociation, cells were plated onto XF24 cell plates (Seahorse Bioscience, Billerica, MA) pre-coated with matrigel, laminin and fibronectin (as above). Optimization of reagents was performed and the protocols and algorithm program were used according to the XF24 analyser. Briefly, cells were transitioned from their oligodendrocyte specific media into the Agilent Seahorse Base Medium which has low buffering capacity and 0 mM glucose and maintained in ambient CO_2_ for 25 min. The plate was then inserted into the machine; baseline oxygen consumption rate and extracellular acidification rate were measured prior to the addition of glucose (10 mM), oligomycin (1.5 μM) and 2-deoxy-glucose (100 mM). Both oxygen consumption rate and extracellular acidification rate were measured three times following the injection of each drug.

### Generation of induced pluripotent stem cells-derived organoids and assessment of myelination

We generated iPSC-derived organoids containing myelinating oligodendrocytes, termed ‘myelinoids’, using a previously published protocol.^27^ Briefly, the protocol mirrors the patterning protocol outlined above; after the selective patterning for oligodendrocytes, myelin induction was initiated by transferring individual spheres onto PTFE-coated cell culture membrane inserts (Millicell) in 6 well plates and cultured at 7.5% CO_2_. Spheres were maintained in this way for 12 weeks using a myelination media supplemented with 1 × ITS, 10 ng/mL IGF-1 and 45 ng/mL T3.

Analyses were conducted on whole-mounted and cryo-sectioned myelinoids. After 12 weeks of culturing in myelination media, myelinoids were fixed in 4% PFA for 2 h before washing and storing in PBS. For immunostaining, myelinoids underwent permeabilization in 0.3% triton-X-100 in PBS for 40 min before blocking in 10% normal goat serum in 0.25% triton-X-100 for 2 h. Antigen retrieval involved incubating myelinoids in citrate buffer (pH 6.0) at 95°C for 20 min followed by incubation in blocking solution again for 1 h. Primary antibodies (CNP, Atlas #AMAB91072, 1:2000; NF-H, Biolegend #822601, 1:10 000; CASPR, Abcam #AB34151, 1:1000) were diluted in blocking solution and incubated overnight at 4°C. Myelinoids were then washed in 0.1% Tween-20 before incubation in appropriate AlexaFluor secondary antibody (all used at 1:1000) for 2 h. Myelinoids were then incubated with DAPI for 10 min, washed in PBS and then mounted onto microscope slides (Thermo Scientific) with FluorSave (Calbiochem) and coverslipped (No, 1.5; Thermo Scientific).

Analyses were conducted as described previously.^27^ Briefly, for manual tracing of individual oligodendrocytes and nearest neighbour analysis, a Zeiss 710 confocal was used to take tiled z-stacks at 40× magnification. The Simple Neurite Tracer plugin was used in FIJI for manual tracing of CNP^+^ myelin sheath lengths per cell, and nearest neighbour analysis was performed by plotting a 100 µm radius circle around individual cells and counting the number of myelinating oligodendrocytes within that area.

### Data availability

All data can be made available upon request to the corresponding authors.

### Statistical analysis

Data are presented as mean ± S.E.M. Molecular, histological and metabolic outputs were analysed using a one-way ANOVA and Bonferroni’s multiple comparisons test or uncorrected Fisher’s least significant difference test for *post* *hoc* comparisons. Sholl data were compared using repeated-measures two-way ANOVA with Bonferroni’s multiple comparisons test for *post* *hoc* comparisons. Rectification indices of passive membrane currents were calculated from the relationship between the conductances of current data at –124 mV and +16 mV using the following equation: *Index* = [*I/*(16 − *E*_REV_)] [*I/*(−124 − *E*_REV_)] where *I* represents current amplitude, and, *E*_REV_ indicates the reversal potential of currents. Statistical comparison of the means was performed by Student’s *t*-test. * indicates *P *<* *0.05; ** indicates *P *<* *0.01; *** indicates *P *<* *0.001. A number of biological and technical replicates are highlighted in figure legends whereby *N* denotes number of independent differentiations and *n* denotes number of cells or number of fields of view analysed.

## Results

### *TARDBP* mutant oligodendrocytes exhibit mutation-dependent TDP-43 cytoplasmic inclusions

**Figure 1:**
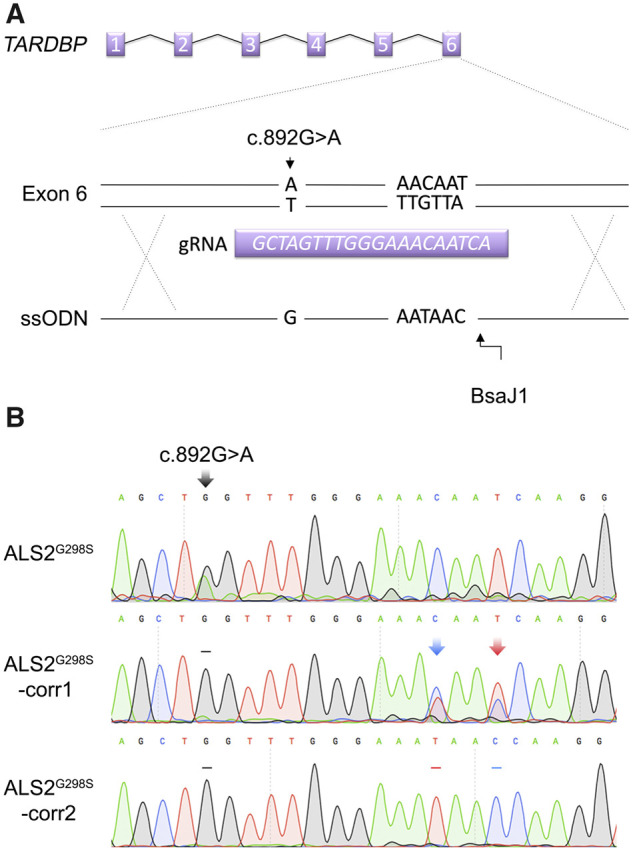
**Gene correction of the G298S mutation**. Using CRISPR-Cas9, the guide RNA (gRNA) and ssODN was designed to insert a guanine at position ORF position 892 to replace an adenine resulting in an amino acid substitution from a glycine to a serine (**A**). The ALS2-corr1 line had a monoallelic substitution and the ALS2-corr2 line had a biallelic substitution (**B**).

We differentiated four iPSC lines (an unrelated control line [control], a patient line with an M337V point mutation in the *TARDBP* gene [ALS^M337V^]) and two clones of a patient line with a G298S point mutation in the *TARDBP* gene [ALS1^G298S^ and ALS2^G298S^] into oligodendrocytes using a method published previously.^22^^,^^25^ We also successfully used CRISPR-Cas9 to gene-edit the ALS2^G298S^ clone and then generated oligodendrocytes from two iPSC clones: one with mono-allelic and other with bi-allelic targeting (ALS2^G298S^-corr1: mono-allelic and ALS2^G298S^-corr2: bi-allelic; Fig.  1).

**Figure 2:**
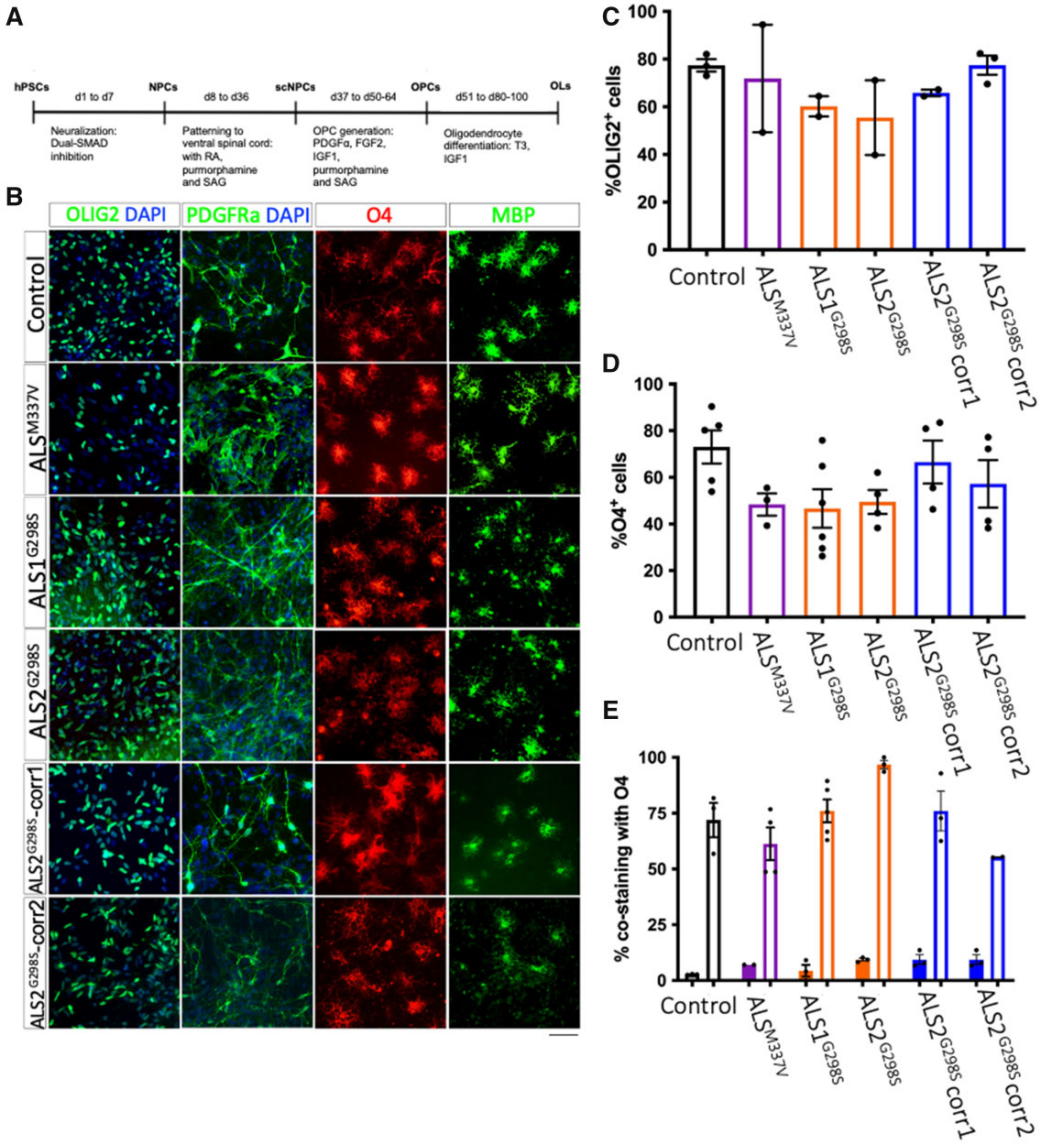
**Oligodendrocytes can be made from iPS lines harbouring a G298S mutation and an M337V mutation in the *TARDBP* gene.** Following a previously published protocol, human-derived pluripotent stem cells were patterned towards the oligodendrocyte lineage; the final step of the process was differentiation from oligodendrocyte precursor cells to mature, post-mitotic oligodendrocytes (**A**). Lines were successfully patterned and markers of the oligodendrocyte lineage are shown in (**B**) for all lines. Scale bar = 50 µm. There was no difference in percentage of Olig2 expressing cells (control *N* = 3, ALS^M337V^ *N* = 2, ALS1^G298S^ *N* = 2, ALS2^G298S^ *N* = 2, ALS2^G298S^-corr1 *N* = 2, ALS2^G298S^-corr2 *N* = 3; *n* = >500 cells counted per line) (**C**) or the percentage of O4 expressing cells (**D**) amongst lines (control *N* = 5, ALS^M337V^ *N* = 3, ALS1^G298S^ *N* = 6, ALS2^G298S^ *N* = 4, ALS2^G298S^-corr1 *N* = 4, ALS2^G298S^-corr2 *N* = 4). There was also no difference in co-expression of O4 and PDGFRα (closed bars; control *N* = 3, ALS^M337V^ *N* = 2, ALS1^G298S^ *N* = 3, ALS2^G298S^ *N* = 3, ALS2^G298S^-corr1 *N* = 3, ALS2^G298S^-corr2 *N* = 3; *n* = 5–10 FOV counted per *N*) nor co-expression of O4 and MBP (open bars; control *N* = 3, ALS^M337V^ *N* = 4, ALS1^G298S^ *N* = 5, ALS2^G298S^ *N* = 3, ALS2^G298S^-corr1 *N* = 3, ALS2^G298S^-corr2 *N* = 2; *n* = 5–10 FOV counted per *N*) between lines 7 days after plate-down (**E**). Data are presented as average ± SEM.

Using a method published previously^22^^,^^25^ (Fig.  2A), all lines efficiently generated oligodendrocytes (Fig.  2B) that expressed Olig2 (Control: 78.04 ± 0.71%; ALS^M337V^: 71.91 ± 7.18; ALS1^G298S^: 59.37 ± 6.27; ALS2^G298S^: 60.69 ± 10.46; ALS2^G298S^-corr1: 72.04 ± 4.61; ALS2^G298S^-corr2: 76.33 ± 3.09; Fig.  2C) and O4 (Control: 73.05 ± 7.10; ALS^M337V^: 48.36 ± 4.85%; ALS1^G298S^: 46.66 ± 4.77; ALS2^G298S^: 49.45 ± 2.96; ALS2^G298S^-corr1: 66.53 ± 6.17; ALS2^G298S^-corr2: 57.23 ± 6.77; Fig.  2D) of which there was co-expression of O4 with MBP (Control: 70.42 ± 12.33; ALS^M337V^: 60.09 ± 12.29%; ALS1^G298S^: 70.36 ± 12.64; ALS2^G298S^: 96.30 ± 2.31; ALS2^G298S^-corr1: 66.61 ± 15.06; ALS2^G298S^-corr2: 55.48 ± 17.06) and co-expression of O4 with PDGFRα (Control: 3.70 ± 4.05; ALS^M337V^: 5.79 ± 5.54%; ALS1^G298S^: 6.18 ± 6.24; ALS2^G298S^: 9.46 ± 8.37; ALS2^G298S^-corr1: 9.27 ± 9.54; ALS2^G298S^-corr2: 9.27 ± 9.54; Fig.  2E).

**Figure 3:**
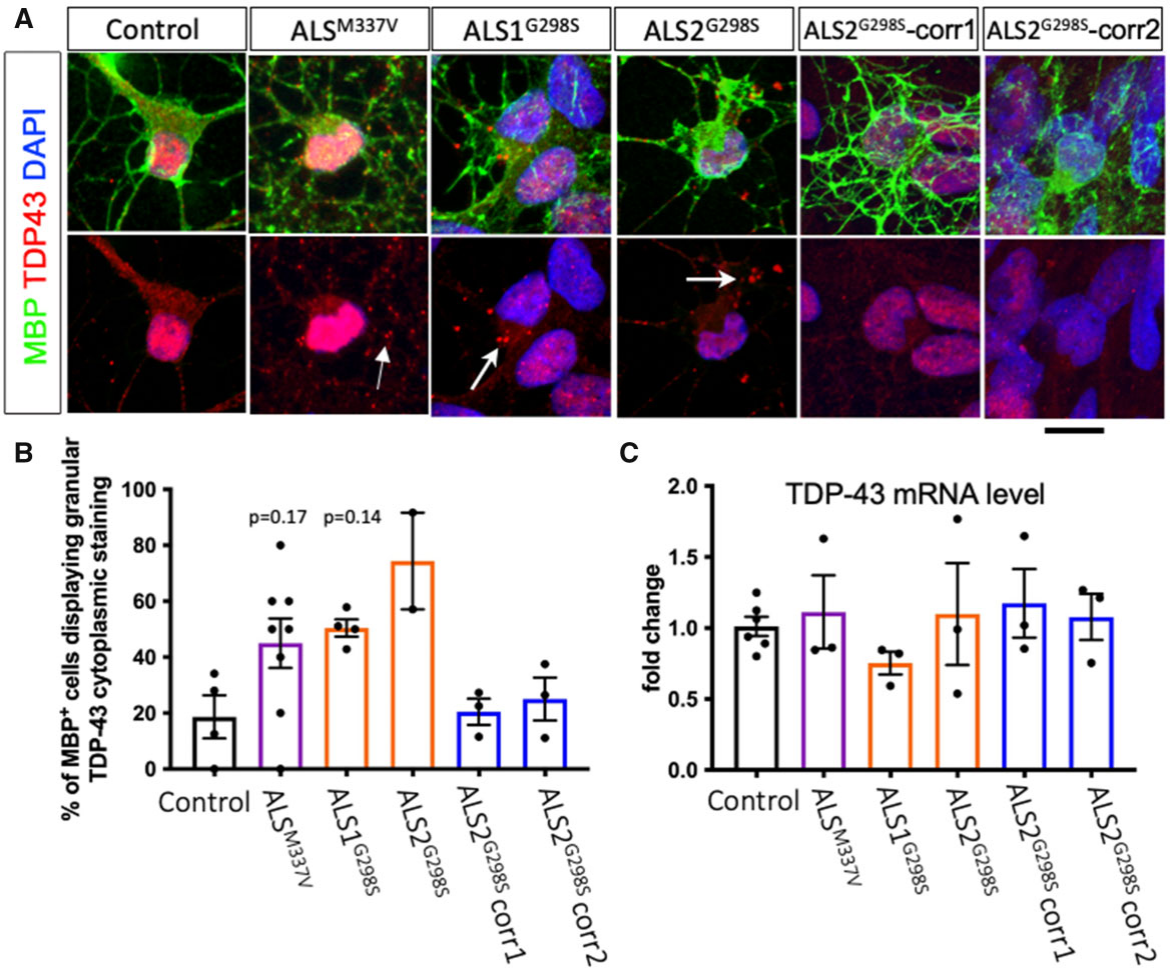
**Point mutations in the *TARDBP* gene causes TDP-43 protein aggregate formation.** The G298S mutation caused TDP-43 cytoplasmic protein aggregations in both ALS^G298S^ clones and the ALS^M337V^ oligodendrocyte and these were not present in the G298S isogenic control lines (**A**). When quantified, there was an increase in MBP^+^ cells with TDP-43 cytoplasmic inclusions in the G298S lines; this was reversed in the isogenic controls (**B**; control *N* = 3, ALS^M337V^ *N* = 7, ALS1^G298S^ *N* = 4, ALS2^G298S^ *N* = 2, ALS2^G298S^-corr1 *N* = 3, ALS2^G298S^-corr2 *N* = 3; *n* = 5–10 FOV counted per *N*). There was no difference in TDP-43 mRNA expression between lines (**C**; control *N* = 6, ALS^M337V^ *N* = 3, ALS1^G298S^ *N* = 3, ALS2^G298S^ *N* = 3, ALS2^G298S^-corr1 *N* = 3, ALS2^G298S^-corr2 *N* = 3). One-way ANOVA and Bonferroni’s multiple comparisons test. Scale bar 12 µm. Data are presented as average ± SEM.

We next examined for evidence of TDP-43 pathology in MBP-positive oligodendrocytes. Both ALS^G298S^ and ALS^M337V^ lines exhibited cytoplasmic mis-accumulation compared to control oligodendrocytes (Fig.  3A and B). Importantly, the cytoplasmic TDP43 inclusions observed in *TARDBP*^G298S^ oligodendrocytes were reverted back to the control levels in the gene-corrected clones (Fig.  3B). Despite the increase in cytoplasmic TDP-43 inclusions, there was no compensatory increase in *TDP-43* mRNA expression (Fig.  3C).

### Cell-autonomous consequences of TDP-43 mutation on oligodendrocyte morphology, electrical activity, metabolic function and myelinating capacity

**Figure 4:**
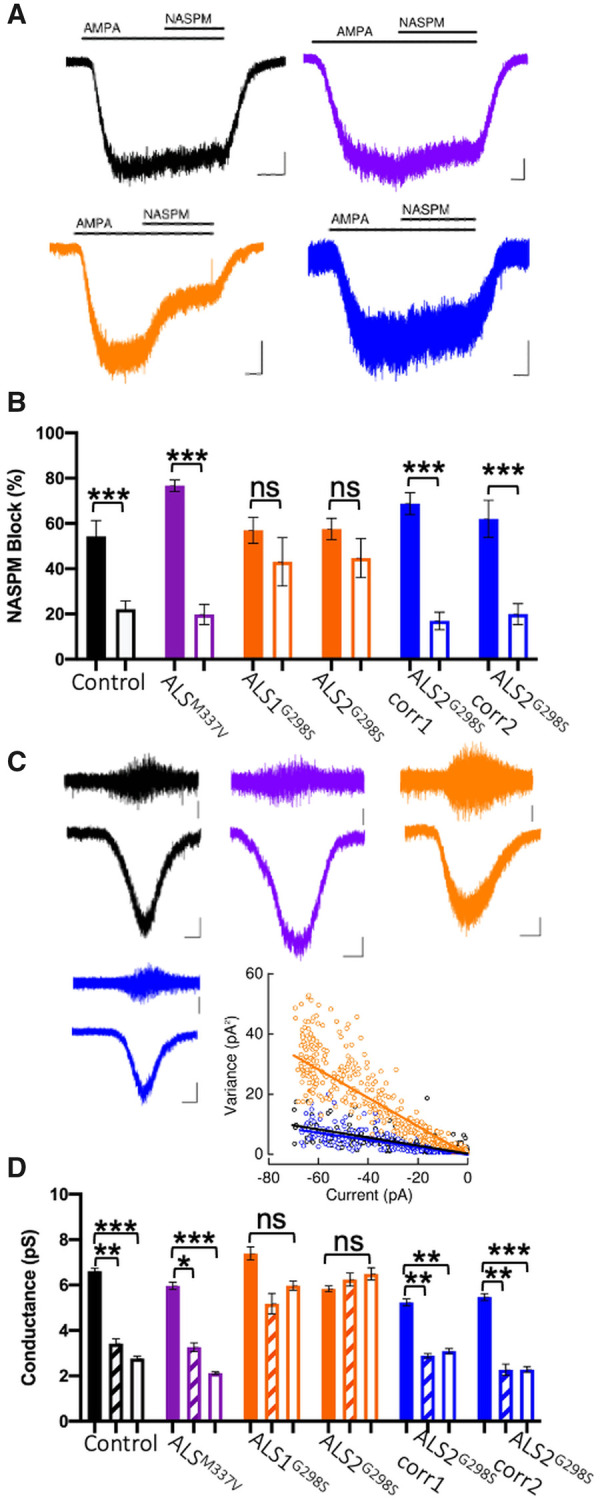
**ALS^G298S^ oligodendrocytes, but not ALS^M337V^ oligodendrocytes exhibit Ca^2+^-permeable AMPARs receptors but this is not due to maturation deficits.** (**A**) Example recording of NASPM block of steady‐state currents evoked by AMPA in week 3 O4^+^‐oligodendrocytes derived from control (black), TDP-43^G298S^ (orange) and TDP-43^G298S^-corr (blue). Scale bar (control) of 25 pA, 5s; scale bar (TDP-43^G298S^) of 50 pA, 10s, and scale bar (TDP-43^G298S^-corr) of 20 pA, 10s. (**B**) Mean (±SEM) percentage NASPM block of AMPA currents in PDGFRα positive oligodendrocyte precursor cells (examined at week 1; solid bars; control *N* = 4 *n* = 12, ALS^M337V^ *N* = 1 *n* = 6, ALS1^G298S^ *N* = 4 *n* = 11, ALS2^G298S^ *N* = 3 *n* = 8, ALS2^G298S^-corr1 *N* = 3 *n* = 10, ALS2^G298S^-corr2 *N* = 2 *n* = 8) and O4 positive oligodendrocytes (examined at week 3; open bars; control *N* = 4 *n* = 19, ALS^M337V^ *N* = 2 *n* = 6, ALS1^G298S^ *N* = 2 *n* = 5, ALS2^G298S^ *N* = 3 *n* = 8, ALS2^G298S^-corr1 *N* = 2 *n* = 9, ALS2^G298S^-corr2 *N* = 3 *n* = 9). (**C**) ALS2^G298S^-corr2 *N* = 2 *n* = 11) Sample nonstationary fluctuation analysis recordings of AMPAR‐mediated currents from week 3 O4^+^‐oligodendrocytes derived from control (black), TDP-43^G298S^ (orange) and TDP-43^G298S^-corr (blue). AC scale bars 10 pA and DC scale bars 20 pA, 5s. Plot describes the linear relationship of the variance of the AC‐coupled current to the DC‐current amplitude for the recordings. The fitted slopes for each plot gave respective unitary single‐channel current amplitude estimates of −0.14, −0.30 and −0.13 pA, respectively, from which the unitary conductance was calculated, (**D**) Mean (± SEM) estimated AMPAR γ in all lines examined in PDGFRα positive oligodendrocyte precursor cells (examined at week 1; solid bars; control *N* = 2 *n* = 12, ALS^M337V^ *N* = 3 *n* = 17, ALS1^G298S^ *N* = 3 *n* = 10, ALS2^G298S^ *N* = 4 *n* = 20, ALS2^G298S^-corr1 *N* = 3 *n* = 15, ALS2^G298S^-corr2 *N* = 3 *n* = 12) and O4 positive oligodendrocytes (examined at week 1; dashed bars; control *N* = 2 *n* = 10, ALS^M337V^ *N* = 2 *n* = 7, ALS1^G298S^ *N* = 2 *n* = 6, ALS2^G298S^ *N* = 2 *n* = 10, ALS2^G298S^-corr1 *N* = 4 *n* = 13, ALS2^G298S^-corr2 *N* = 1 *n* = 4, and week 3; open bars; control *N* = 2 *n* = 12, ALS^M337V^ *N* = 4 *n* = 17, ALS1^G298S^ *N* = 3 *n* = 14, ALS2^G298S^ *N* = 3 *n* = 13, ALS2^G298S^-corr1 *N* = 4 *n* = 13.

Glutamate receptor dysregulation is a major hypothesis driving ALS pathogenesis. This study is the first to examine AMPAR dysregulation in oligodendrocytes harbouring mutations in the *TARDBP* gene (Fig.  4).

We assessed the proportion of Ca^2+^-permeable AMPARs in OPCs (PDGFRα^+^ cells) and mature oligodendrocytes (O4^+^/PDGFRα^−^ cells) by measuring the block of AMPA-mediated currents by Ca^2+^-permeable AMPAR channel blocker 1-naphthyl acetyl spermine (Fig.  4A).^26^ It was found that upon differentiation of OPC to mature oligodendrocytes, there was the normal transition from Ca^2+^-permeable to Ca^2+^-impermeable AMPAR in control (black bars) and ALS^M337V^ (purple bars). In contrast, the ALS^G298S^ oligodendrocytes maintain functional expression of Ca^2+^-permeable AMPARs (Fig.  4A and B; orange bars). Importantly, this phenotype was rescued in ALS^G298S^-corr oligodendrocytes with the level of 1-naphthyl acetyl spermine block mirroring control and ALS^M337V^ oligodendrocytes, consistent with Ca^2+^-impermeable AMPARs (Fig.  4A and B).

To assess whether this dysregulation was due to delayed maturation of AMPAR composition, we examined the unitary conductance of AMPARs in each lines by assessing non-stationary fluctuation analysis of AMPA-mediated currents (Fig.  4C and D), as performed previously.^22^^,^^23^^,^^26^ We determined that the differentiation of PDGFRα-positive OPC to O4-positive oligodendrocytes was associated with a significant shift from 6.60 ± 0.14 to 3.42 ± 0.21 pS in the control line (Fig.  4D), reproducing earlier data.^22^ These data are consistent with a Ca^2+^-permeable to Ca^2+^-impermeable switch in AMPAR composition. In contrast, the ALS^G298S^ clones display persistently high unitary single channel conductance in week 3 oligodendrocytes (ALS1^G298S^: OPC data 7.39 ± 0.29 pS, OL data 5.18 ± 0.45 pS; ALS2^G298S^: OPC data 5.83 ± 0.13 pS, OL data 6.24 ± 0.29 pS; Fig.  4D), consistent with an increased Ca^2+^-permeability. Importantly, this phenotype is rescued in the ALS^G298S^-corr lines (blue bars) implying a genotype-specific AMPAR phenotype in oligodendrocytes harbouring a *TARDBP^G298S^* mutation. Surprisingly, ALS^M337V^ oligodendrocytes did not show an equivalent dysregulation (OPC data 5.96 ± 0.16 pS, OL data 3.26 ± 0.19 pS; Fig.  4D). Therefore, the dysregulation in AMPAR was specific to the G298S genotype, and its persistence through to 3-week-old oligodendrocytes suggests it was not due to delayed maturation of AMPAR composition in oligodendrocytes.

To further verify that the differences in AMPAR were not due to impairments in maturation in TDP-43^G298S^ oligodendrocytes, we next assessed the membrane properties of the cells.^22^ We found no correctable difference in cell capacitance (Supplementary Fig. 1A and B), nor membrane resistance (Supplementary Fig. 1C) or rectification of membrane currents (Supplementary Fig. 1D), indicating that the AMPAR dysregulation was not a result of impaired maturation of oligodendrocytes. Further, we also demonstrate no difference in oligodendrocyte morphology with no correctable difference in cell size or number of processes measured using Sholl analysis (Supplementary Fig. 1E) indicating that the AMPAR dysregulation was also not a result of changes to cell morphology.

**Figure 5:**
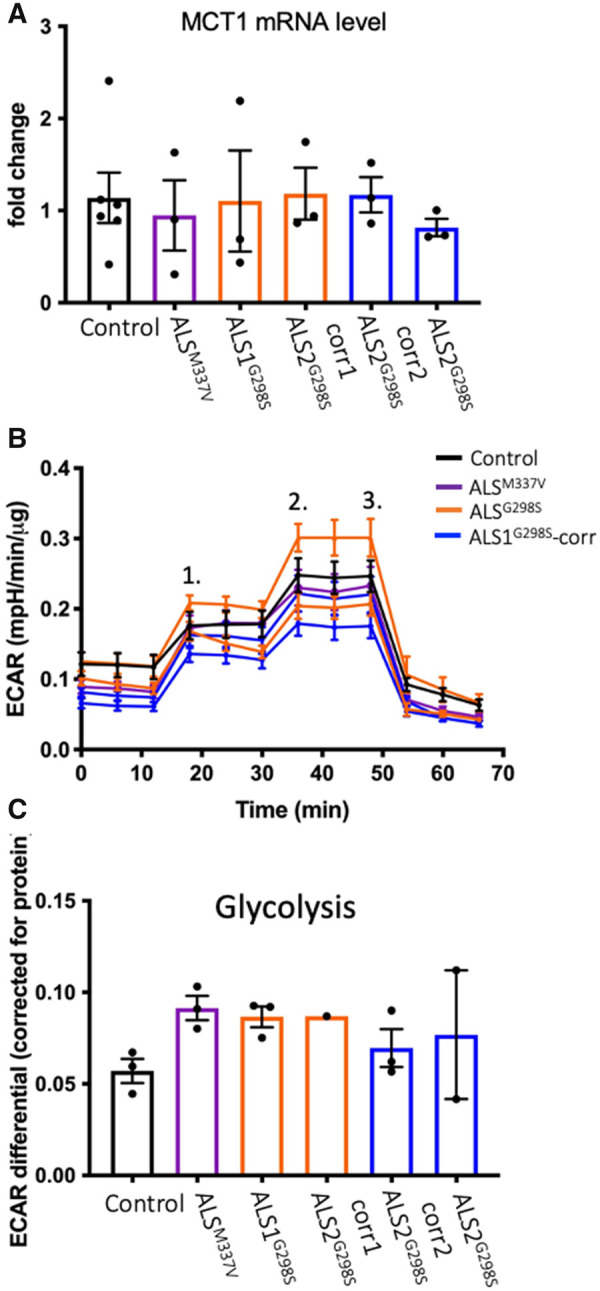
**The G298S and M337V mutations have no effect on oligodendrocyte metabolism.** The G298S and M377V mutations cause no change to mRNA expression of MCT1 (**A**; control *N* = 6, ALS^M337V^ *N* = 3, ALS1^G298S^ *N* = 3, ALS2^G298S^ *N* = 3, ALS2^G298S^-corr1 *N* = 3, ALS2^G298S^-corr2 *N* = 3). MCT1 is responsible for shuttling lactate out of oligodendrocytes; lactate is a principal energy supply for neurons. Using the Agilent Seahorse Analyzer, the ECAR was successfully measured in all lines; they all had appropriate responses to glucose (1.), oligomycin (2.) and 2-deoxy-glucose (3.) (**B**; control *N* = 3 *n* = 2–3 wells per *N*, ALS^M337V^ *N* = 3 *n* = 3–4 wells per *N*, ALS1^G298S^ *N* = 3 *n* = 4–8 wells per *N*, ALS2^G298S^ *N* = 1 *n* = 4 wells, ALS2^G298S^-corr1 *N* = 3 *n* = 3–6 wells per *N*, ALS2^G298S^-corr2 *N* = 3 *n* = 3–6 wells per *N*). There was no difference in glycolysis between lines (**C**; control *N* = 3 *n* = 2–3 wells per *N*, ALS^M337V^ *N* = 3 *n* = 3–4 wells per *N*, ALS1^G298S^ *N* = 3 *n* = 4–8 wells per *N*, ALS2^G298S^ *N* = 1 *n* = 4 wells, ALS2^G298S^-corr1 *N* = 3 *n* = 3–6 wells per *N*, ALS2^G298S^-corr2 *N* = 2 *n* = 3–6 wells per *N*). Data are presented as average ± SEM.

Oligodendrocytes are increasingly recognized to provide metabolic support to neurons with some evidence for dysregulation in ALS.^14^^,^^28–30^ To determine if there was a mutant-dependent effect on metabolic function, we first measured expression of the key lactate transporter *MCT1* and found no difference between control and mutant lines (Fig.  5A). Next, we used the Agilent Seahorse XF Analyzer to assess changes in extracellular acidification rate representative of basal glycolysis, maximum glycolytic capacity and glycolytic reserve of oligodendrocytes by addition of glucose, oligomycin and 2-deoxy-glucose, respectively. No difference between any of the lines at any of the assay timepoints was observed (Fig.  5B). Quantification of the gradient shift in extracellular acidification rate after addition of glucose (representing basal glycolysis) was not different between genotypes (Fig.  5C).

To evaluate whether mutations in the *TARDBP* gene impaired an oligodendrocyte’s capacity to myelinate, we used a recently reported organoid system^27^ that reproducibly yields widespread and compact myelin with nodal organization. Given the more severe phenotype in the G298S oligodendrocytes, we chose to focus on the ALS2^G298S^ patient line and its CRISPR-Cas9 gene-edited control line ALS2^G298S^-corr1. We observed widespread myelination in our 3D organoid model derived from both ALS2^G298S^ and ALS2^G298S^-corr1 isogenic line (representative image of an ALS2^G298S^ myelinating organoid in Supplementary Fig. 2A and higher magnification images of myelinating oligodendrocytes from ALS2^G298S^ and ALS2^G298S^-corr1 in Supplementary Fig. 2B) with intact paranode formation as shown by the presence of CASPR at the distal ends of myelin sheaths (Supplementary Fig. 2C). We also noted no statistical difference in oligodendrocyte density between the two cell lines (Supplementary Fig. 2D). Next, by manually tracing sheaths of individual oligodendrocytes, we found no difference in the number or mean length of internodes produced by each oligodendrocyte (Supplementary Fig. 2D and E). The frequency distribution of internode lengths in G298S oligodendrocytes was also unaffected (Supplementary Fig. 2F). Taken together, these results demonstrate that the *TARDBP* mutation has no effect on myelin formation.

## Discussion

We report the demonstration of TDP-43 cytoplasmic mis-localization and selective genotype-dependent evidence of AMPAR dysregulation in ALS patient iPSC-derived oligodendrocytes. These findings are consistent with both previous human autopsy studies characterizing TDP-43 in sporadic ALS cases^12^^,^^15^ and confirm recapitulation of TDP-43 proteinopathy in both motor neurons and astrocytes derived from TDP-43 mutant iPSC lines^24^^,^^31^ and astrocyte cultures.^32^

Despite oligodendrocytes exhibiting TDP-43 protein mis-localization, there was no demonstrable deficit on maturation, morphology, metabolic function or myelination. We did find an effect on AMPAR properties implying a potential contributing role of oligodendrocytes to the excitotoxicity phenotype characteristic of ALS. The literature implicating oligodendrocytes in ALS is brief, but growing, and spans human post-mortem tissue,^11^^,^^13^^,^^15^ mouse models,^13^^,^^15^ and is only beginning to be modelled using iPSC.^33^ Collectively, it suggests a cell autonomous and non-cell autonomous deficit in oligodendrocytes in ALS. Our electrophysiological data suggesting oligodendrocytes harbouring a G298S point mutation in the *TARDBP* gene, but not an M337V mutation, retain Ca^2+^-permeable AMPAR is of interest. AMPAR dysregulation in genetic and sporadic ALS has previously been demonstrated in neurons^21^^,^^23^^,^^34^ but our data are the first to implicate oligodendrocytes, albeit with genotype specificity. Indeed, similar to our data, the G298S genotype has previously been associated with a more severe phenotype than the M337V genotype in both fly^35^ and mouse studies^36^ therefore highlighting ALS heterogeneity, even between point mutations within the same gene. To account for these differences, there remains a need for future studies of multiple additional lines (with isogenic controls). Through our comprehensive assessment of oligodendrocyte generation and development (Fig.  2), we found no correctable differences in the oligodendrocyte density suggesting the AMPAR dysregulation did not lead to a baseline survival deficit. Given the mixed population generated during oligodendrocyte generation *in vitro*, it remains challenging to assess subsequent excitotoxicity as a result of AMPAR dysregulation in our cultures. Our data therefore validate the importance of considering oligodendrocytes in the context of unravelling the contribution of excitotoxicity to ALS pathology, a relationship that is already established in demyelinating diseases. Indeed, the deletion of AMPARs specifically from oligodendrocytes in mice has been shown to not only prevent demyelination but also preserve axonal integrity.^37^ Investigating this phenomenon further in an ALS context by utilizing co-culture systems and *in vivo* models is imperative.

The absence of deficits in glycolytic function is in contrast to findings from Ferraiuolo and colleagues^33^ who found that iPSC-derived oligodendrocytes with a TDP-43 mutation produce less lactate than comparative controls. This difference could be attributed to the difference in the challenge to the cells; Ferraiuolo and colleagues measured basal lactate concentrations whereas the Seahorse assay is a glycolytic stress test so has measured the cells’ ability to undergo glycolysis whilst under stress. Further, our patterning protocol produces a cell population that contains a proportion of astrocytes. Both studies focus on pre-myelinating oligodendrocytes, so more sophisticated and/or longer-term assays of oligodendrocyte behaviour that allow assessment of myelin generation and maintenance are required to reveal the subsequent deleterious effects of a TDP-43 mutation in oligodendrocyte lineage cells on both myelination as well as metabolic capacity. This is particularly important given that despite we, and others, showing no change to MCT1 expression in iPSC-derived oligodendrocytes harbouring *TARDBP* mutations,^33^ in end-stage mouse and human disease MCT1 protein levels were found to be decreased.^13^^,^^14^ Myelinating organoid experiments revealed no effect of a *TARDBP* mutation on myelination and the generation of myelin internodes. This is consistent with a recent rodent study that found no effect of selective TDP-43 deletion in oligodendrocytes on paranodal assembly.^38^ Further studies are needed to ascertain myelination status and the influence of *TARDBP* mutation over time to model prolonged disease.

In summary, we report recapitulation of TDP-43 pathology in oligodendrocytes that is TDP-43 mutation dependent establishing a platform for further studies in the arena of both disease modelling and drug discovery. Further, we identified a genotype-specific AMPAR phenotype highlighting a cell autonomous impairment in oligodendrocytes that emphasizes the importance of considering oligodendrocytes as a key player in ALS pathogenesis.

## Supplementary material

Supplementary material is available at *Brain Communications* online.

## Supplementary Material

fcab255_Supplementary_Data

## Abbreviations

ALS =: amyotrophic lateral sclerosis
AMPAR =: α-amino-3-hydroxy-5-methyl-4-isoxazolepropionic acid receptor
DAPI =: 4′,6-diamidino-2-phenylindole
ECAR =: extracellular acidification rate
FOV =: fields of view
iPSC =: induced pluripotent stem cell
MBP =: myelin basic protein
NASPM =: 1-naphthyl acetyl spermine
O4 =: oligodendrocyte marker O4
OCR =: oxygen consumption rate
OLIG2 =: oligodendrocyte transcription factor 2
OPC =: oligodendrocyte precursor cell
PBS =: phosphate buffered saline
PDGF =: platelet derived growth factor
TDP-43 =: transactive response DNA-binding protein-43
