# Supplementary figures and images for: Transactive response DNA-binding protein-43 proteinopathy in oligodendrocytes revealed using an induced pluripotent stem cell model
Source: Brain Commun. 2021 Oct 26;3(4):fcab255. doi: 10.1093/braincomms/fcab255 (PMC8936427; doi:10.1093/braincomms/fcab255)

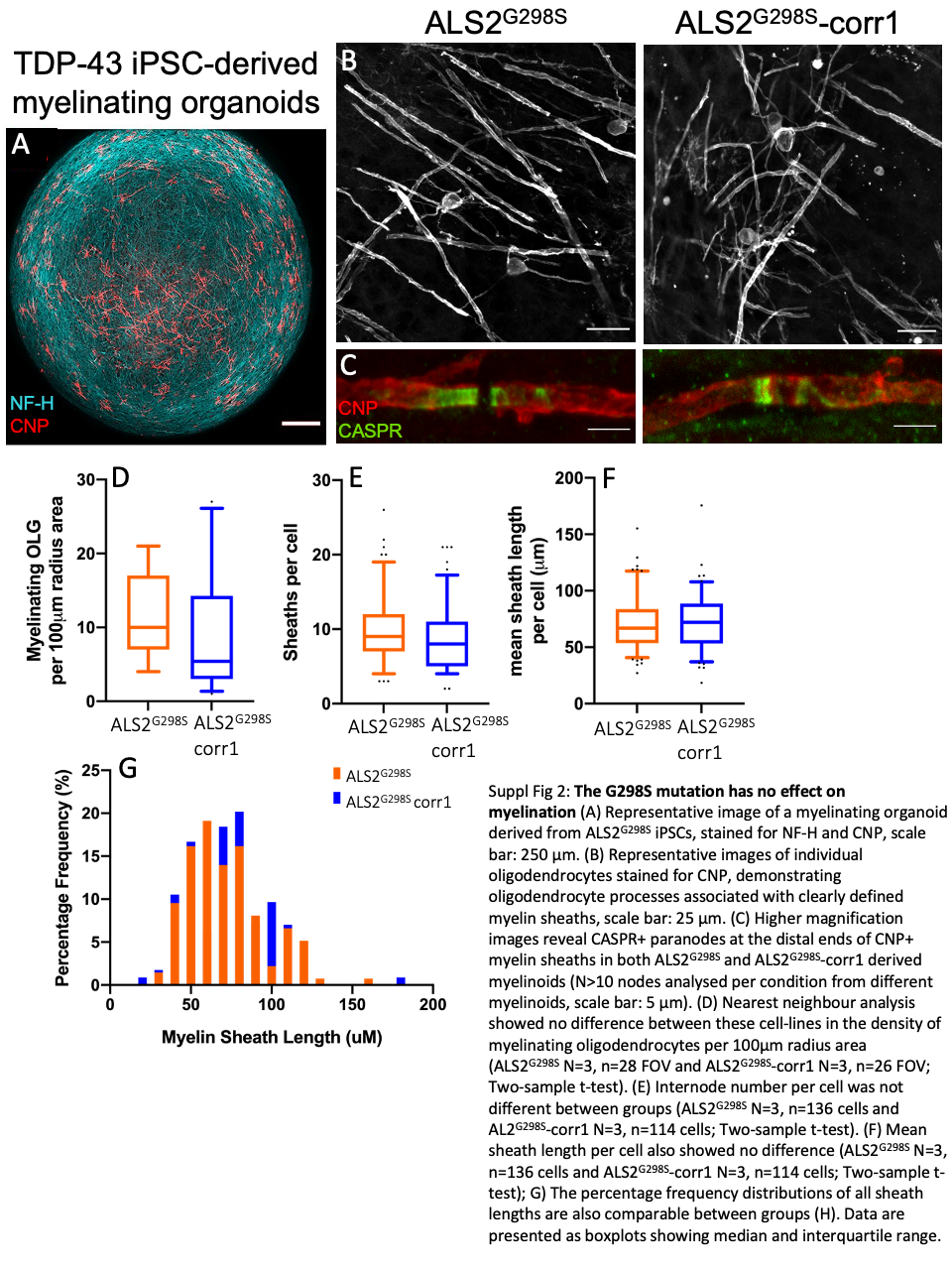

Supplement: fcab255_Supplementary_Data [file fcab255_Supplementary_Data.zip › Supplemental Fig 2.tiff]

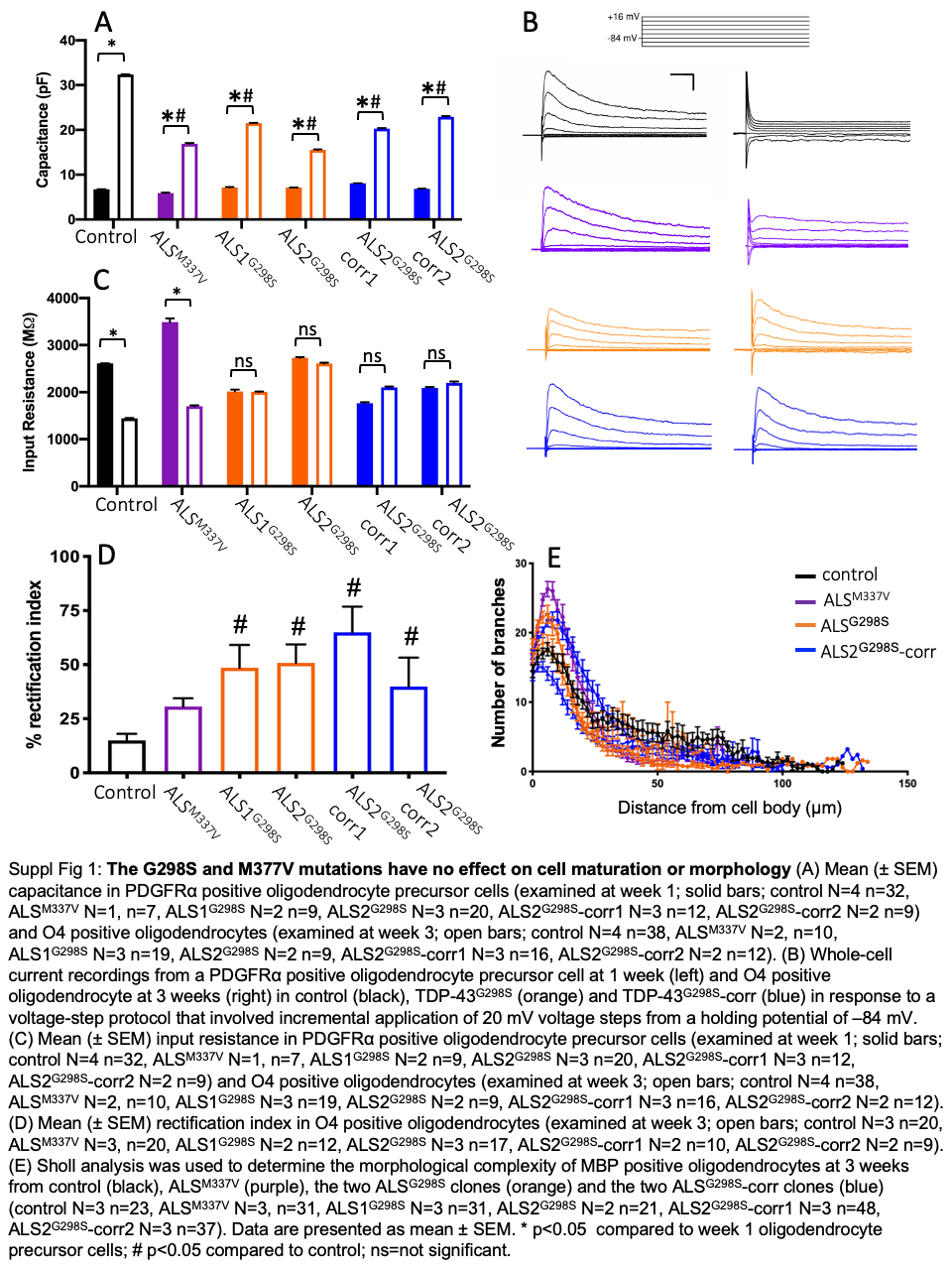

Supplement: fcab255_Supplementary_Data [file fcab255_Supplementary_Data.zip › Supplemental Fig 1.tiff]
